# Workplace and community social capital and burnout among professionals of health and welfare services for the seniors: A multilevel analysis in Japan

**DOI:** 10.1002/1348-9585.12177

**Published:** 2020-10-31

**Authors:** Hiroshi Murayama, Kumiko Nonaka, Masami Hasebe, Yoshinori Fujiwara

**Affiliations:** ^1^ Research Team for Social Participation and Community Health Tokyo Metropolitan Institute of Gerontology Tokyo Japan

**Keywords:** burnout, Japan, multilevel analysis, professional, social capital

## Abstract

**Objective:**

Despite the potential of the social capital approach in preventing burnout, there is sparse evidence of its contextual effect. This study aimed to reveal the contextual association of workplace and community social capital on burnout among professionals of health and welfare services for seniors in Japan.

**Methods:**

We collected data from a cross‐sectional questionnaire survey for all health and welfare professionals working in Community Comprehensive Support Centers (CCSCs) in the central Tokyo area in 2015. We assessed burnout using the Japanese version of the Maslach Burnout Inventory, which consists of three subscales: emotional exhaustion, depersonalization, and reduced personal accomplishment. We prepared social capital items regarding workplace (the CCSC the participants belonged to) and community (the current catchment area of the CCSC). We aggregated individual responses of workplace and community social capital within each CCSC to create group‐level workplace and community social capital indicators.

**Results:**

Among the 1771 questionnaires distributed, we analyzed 1110 from 211 CCSCs. Multilevel analysis showed that higher group‐level workplace social capital was significantly associated with lower scores of all three subscales after adjusting for covariates. Moreover, we found a significant association between greater group‐level community social capital and lower scores of depersonalization and reduced personal accomplishment.

**Conclusion:**

Working in workplaces and communities with higher social capital is related to lower burnout. The findings suggest that strategies to enhance the social capital of their workplace and community would be beneficial in the prevention of burnout among professionals in the field of health and social welfare.

## 1 INTRODUCTION

Prevention of burnout is an important strategy for the administration of healthcare and welfare organizations. According to the definition by Maslach and Jackson,^1^ burnout is a multidimensional, psychological syndrome of emotional exhaustion, depersonalization, and reduced personal accomplishment that can occur among individuals who work with other people in some capacity. Burnout consists of three dimensions: “emotional exhaustion,” which indicates feelings of being emotionally overextended and exhausted at one's work; “depersonalization,” which refers to an unfeeling emotional state and impersonal response toward recipients of one's service, care, treatment, or instruction; and “reduced personal accomplishment,” which signals negative feelings of competence and successful achievement in one's work with people. Previous studies reported that burnout could have adverse effects on workers’ health such as mortality,^2^ coronary heart disease,^3^ depressive symptoms,^4^, ^5^ and insomnia.^6^ Other studies have shown that burnout can also effect organizational management through long‐term sick absence^7^ and presenteeism,^8^ and job dissatisfaction.^9^ Similar trends in the effects of burnout were observed among professionals in health and welfare services.^10^, ^11^, ^12^, ^13^, ^14^, ^15^, ^16^, ^17^

To determine the appropriate clinical strategy to prevent burnout in the field of health and social welfare, it is essential to understand its risk factors among health and welfare professionals. Systematic reviews addressed two types of relevant factors of burnout.^18^, ^19^, ^20^, ^21^, ^22^ These were personal variables (eg age, sex, marital status, socioeconomic status, personality traits, and coping strategy) and work‐related variables (eg job characteristics, workload, job control, job demands, and workplace support). Besides, another work‐related factor associated with burnout can be social capital.

To date, many studies have explored the association between social capital and health. Kawachi and Berkman defined social capital as resources that are available to individuals because of their membership in a network or a group.^23^ Social capital is generally classified as “individual‐level” or “group‐level.” At the individual level, social capital refers to resources embedded within an individual's social networks; that is, it is regarded as a property of individuals. In contrast, at the group level, social capital represents the resources available to members of tightly knit communities. This tends to emphasize social capital as a group attribute that can be analyzed as a contextual influence on individual health.

A number of studies have reported the association between higher workplace social capital and less likelihood of burnout,^24^, ^25^, ^26^, ^27^ but they focused on individual‐level social capital. That is, they dealt with social capital as individuals’ perceptions of cohesion and solidarity in the workplace, not as attributes of the workplace where people belong. When developing strategies utilizing social capital to prevent burnout at the workplace level, it is essential to test the effect of social capital as the workplace attribute on burnout.

Moreover for professionals in the field of health and social welfare, social capital of the community where they are working could influence their burnout status. For example, higher community social capital can increase access to healthcare services^28^; professionals working in such communities might be able to feel more rewarded and prouder of their activities. In addition, negative feedback (eg violence) from clients/patients to professionals leads to burnout.^29^ As cohesive communities can provide ease and relief to local residents through dense support systems,^23^ professionals working in such communities might receive less negative feedback resulting in less burnout. Therefore, community social capital is possibly an important factor affecting professionals’ burnout.

To prevent burnout, much attention has been paid to job redesign interventions focusing on the increase of task variety and the decrease of job demand. Given that the association between greater social capital in the workplace and community and lower level of burnout was observed, we suggest that working environment redesign, including enhancing social interaction and increasing the levels of trust, reciprocity, and support within the workplace and community, would be an effective strategy for burnout prevention among professionals engaged in health and welfare services.

Japan has Community Comprehensive Support Centers (CCSCs), which function as hubs for the provision of health and welfare for the seniors in the community. The CCSCs were established in 2006 throughout the country in response to the rapidly aging population, and exist in all local municipalities (5079 centers as of April 2018). They are multidisciplinary organizations in which three professionals (public health nurses/registered nurses, certified social workers, and care managers/care workers) work together to provide support to older people.

According to previous studies from Japan, professionals working in the CCSCs had burnout as severe as other healthcare professionals, and negative feelings toward the CCSC activity (eg socioemotional rewarding) and workload were associated with burnout.^30^, ^31^ However, to the best of our knowledge, there has been no study examining the association between social capital (workplace‐level and community‐level) and burnout among the CCSC workers. Therefore, this study examined the contextual associations of workplace and community social capital with burnout among professionals working at the CCSCs in Japan.

## 2 METHODS

### 2.1 Participants and settings

We conducted a cross‐sectional survey in February and March 2015, using a mailed, self‐administered questionnaire. The target population consisted of professionals belonging to CCSCs of the 23 Wards in the central Tokyo area. As of January 2015, the population in the Tokyo 23 Wards was 9,102,598, and the proportion of people aged ≥65 years was 21.5%.

There were 286 CCSCs in the Tokyo 23 Wards, as of January 2015. Among them, 10 were directly managed by local government (ie the CCSC members were public servants), and the other 276 were consigned by the government to nongovernment organizations (eg medical corporations, social welfare service corporations, and private companies) and managed by them. Because the Ministry of Health, Labour and Welfare states the different roles and functions of local government‐managed CCSCs and nongovernment‐managed CCSCs, the situations surrounding the CCSC staff, including their tasks and responsibilities as well as their employment treatment, are different between these two types of CCSCs. Therefore, we excluded the 10 government‐managed CCSCs from the survey. Consequently, 1771 people working at 276 CCSCs were included in the study. The questionnaires were mailed directly to the CCSCs.

The study protocol was reviewed and approved by the Ethical Committee of the Tokyo Metropolitan Institute of Gerontology. All participants were informed about the purpose of this study and that their participation was optional before inclusion in the study. This statement, a guarantee of anonymity, and other aspects of the cooperation requested were attached to the questionnaire. Return of the questionnaire was viewed as consent to participate in the survey.

### 2.2 Measures

#### 2.2.1 Burnout

We assessed burnout using the Japanese version of the Maslach Burnout Inventory (J‐MBI), consisting of 17 items.^32^ The J‐MBI measures three dimensions of burnout: “emotional exhaustion” (5 items), “depersonalization” (6 items) and “reduced personal accomplishment” (6 items). Respondents answered each question using a 5‐point scale (“1 = none,” “2 = rarely,” “3 = sometimes,” “4 = often,” or “5 = always”). We summed up the answers for each subscale (possible score range: 5‐25 for emotional exhaustion and 6‐30 for depersonalization and reduced personal accomplishment). Higher scores indicated more severe burnout status. Cronbach's alphas in this study were 0.81 for emotional exhaustion, 0.82 for depersonalization, and 0.77 for reduced personal accomplishment, respectively.

#### 2.2.2 Social capital

We prepared social capital items regarding the workplace (ie the CCSC the participants belonged to) and community (ie the current catchment area of the CCSC) where the participants belonged, based on the previous studies.^23^, ^33^ Regarding workplace social capital, we asked the participants six items. These included “The workplace has a positive and friendly atmosphere,” “The workplace is cohesive,” “Communication among the members at the workplace is smooth,” “The members at the workplace can discuss and exchange their opinions actively,” “The members can consult with their colleagues about their daily business,” and “The members at the workplace help each other in a busy time.” Besides, the participants were asked their perceptions in terms of community social capital using five items: “The residents are cohesive,” “The residents trust each other,” “The residents greet each other,” “The residents usually help each other,” and “The residents frequently interact with their neighbors.” Respondents answered these items using a 7‐point Likert scale (“1 = strongly disagree,” “2 = disagree,” “3 = slightly disagree,” “4 = neither,” “5 = slightly agree,” “6 = agree,” or “7 = strongly agree”). We summed up the items for workplace social capital and community social capital, respectively (possible score range: 6‐42 for workplace social capital and 5‐35 for community social capital). Higher scores indicated higher social capital.

In the data set, participant individuals were nested within the CCSC they belonged to. Therefore, we created the workplace and community social capital indicators by aggregating individual responses by the CCSC and calculating the average scores of workplace and community social capital among the respondents in each CCSC, respectively (hereafter, we call these as “group‐level workplace social capital” and “group‐level community social capital”).

#### 2.2.3 Covariates

Age (“20‐29 years,” “30‐39 years,” “40‐49 years,” “50‐59 years,” or “≥ 60 years”), sex (“men” or “women”), educational attainment (“junior high school graduate,” “high school graduate,” “junior college/vocational school graduate,” or “college/graduate school graduate”), self‐rated health (“good” or “poor”), and sense of coherence (SOC) were measured as the respondent individuals’ characteristics. The 3‐item SOC scale assessed SOC, which is a concept that reflects the ability to cope with stress.^34^ Respondents answered the items using a 7‐point Likert scale. The score ranged from 3 to 21, and a higher score represents greater SOC (Cronbach's α = 0.84). We assessed 5 CCSC‐related variables: employment status (“full‐time” or “part‐time”), tenure in a managerial position in the CCSC (“yes” or “no”), years of experience in the current catchment area (“1‐3 years,” “4‐6 years,” or “≥7 years”), type of profession (“public health nurse/registered nurse,” “certified social worker,” or “care manager/care worker”), and average weekly working hours (“<30 hours,” “30‐39 hours,” “40‐49 hours,” “50‐59 hours,” “60‐69 hours,” or “≥70 hours”).

In addition, we used the proportion of people aged ≥65 years in the catchment area of the CCSC as a group‐level variable because this could be a confounder of the association between group‐level social capital (particularly community social capital) and burnout. We captured this information from the questionnaire.

### 2.3 Statistical analysis

To examine the associations between group‐level workplace and community social capital and the subscales of the J‐MBI, we fitted a multilevel model that included a random intercept to the data. We performed the estimation using the full information maximum likelihood procedure. Individual‐level indicators were centered on the group mean and group‐level indicators were centered on the grand mean in order to overcome the problem of collinearity between individual‐level and group‐level variables.

We adopted the following modeling strategy. In Model 1, each individual‐level variable including individual perception of workplace and community social capital was included. In Model 2, the group‐level variables (ie workplace and community social capital, and the proportion of people aged ≥65 years in the catchment area) were added to Model 1.

The results of the fixed effect appear as unstandardized regression coefficients with standard errors. The results of random effects appear as individual‐level and district‐level random variances and intra‐class correlations. A statistical significance was set as *P* < .05. For all analyses, we used HLM 8 (Scientific Software International, Inc, Skokie, IL, USA).

## 3 RESULTS

Of the 1771 questionnaires distributed to 276 CCSCs, 1174 from 248 CCSCs returned (response rate: 66.3%). We excluded 2 questionnaires from respondents that did not identify the belonging CCSC and 62 questionnaires from the participants of the 37 CCSCs that included two or fewer respondents. In total, we analyzed 1110 questionnaires, which included data from 211 CCSCs that had three or more respondents. The average number of the respondents nested in each CCSC was 5.3, ranging from 3 to 12.

Table 1 shows the characteristics of the study participants. The age subgroup of 40‐49 years was the most popular in the sample (35.8%), and 76.8% were women. More than half of the sample graduated from college or graduate school (56.2%). A total of 87.1% of the participants were full‐time workers, and 49.6% had worked 1‐3 years in the current catchment area of the CCSC where they belonged. With regard to the type of profession, 20.3%, 33.1%, and 46.6% were public health nurses/registered nurses, certified social workers, and care managers/care workers, respectively.

**TABLE 1:** Characteristics of the participants (n = 1110)

	n	%	Mean ± SD
Age (y)			
20‐29	71	6.4	
30‐39	272	24.5	
40‐49	397	35.8	
50‐59	290	26.1	
≥60	79	7.1	
Sex			
Men	257	23.2	
Women	852	76.8	
Educational attainment			
Junior high school graduate	85	7.7	
High school graduate	290	26.4	
Junior college/vocational school graduate	107	9.7	
College/graduate school graduate	618	56.2	
Self‐rated health			
Good	934	85.1	
Poor	164	14.9	
Sense of coherence (possible range: 3‐21)			14.6 ± 3.2
Employment status			
Full‐time	966	87.1	
Part‐time	143	12.9	
Managerial position			
Yes	106	9.6	
No	1002	90.4	
Years of experience in the current catchment area of the CCSC (y)			
1‐3	552	49.6	
4‐6	269	24.2	
≥7	290	26.1	
Type of profession			
Public health nurse/registered nurse	221	20.3	
Certified social worker	360	33.1	
Care manager/care worker	508	46.6	
Average weekly working hours (h)			
<30	41	3.8	
30‐39	241	22.1	
40‐49	676	62	
50‐59	110	10.1	
60‐69	13	1.2	
≥70	9	0.8	
Workplace social capital (possible range: 6‐42)			30.1 ± 7.1
Community social capital (possible range: 5‐35)			21.0 ± 4.5
Emotional exhaustion (possible range: 5‐25)			13.0 ± 4.6
Depersonalization (possible range: 6‐30)			10.6 ± 4.1
Reduced personal accomplishment (possible range: 6‐30)			21.4 ± 4.1

Note

Missing value was removed.

Abbreviations: CCSC, Community Comprehensive Support Center; SD, standard deviation.

Table 2 illustrates the characteristics of the group‐level variables and correlations among the variables. The correlation between workplace social capital and community social capital was 0.13.

**TABLE 2:** Characteristics of the group‐level variables and their correlations (n = 211)

	Mean ± SD	Min‐Max	Correlation coefficient (Pearson's *r*)		
			a	b	c
a. Workplace social capital	29.9 ± 5.2	12.3‐39.5		0.13	−0.01
b. Community social capital	21.0 ± 2.9	11.5‐29.0			0.17
c. Proportion of people aged ≥ 65 y in the catchment area of the CCSC (%)	22.0 ± 3.5	12.6‐36.4			

Note

Abbreviations: CCSC, Community Comprehensive Support Center; SD, standard deviation.

Table 3 indicated the results of multilevel analysis to examine the association between group‐level workplace and community social capital and the three subscales of the J‐MBI. In Model 1, we first included only individual‐level variables as fixed effects. Individual‐level workplace social capital was associated with lower scores of all subscales, while individual‐level community social capital was only related to a lower score of reduced personal accomplishment. A higher score of SOC had a significant association with lower scores of all subscales. Being male, of older age, and having better self‐rated health and full‐time employment were related to lower emotional exhaustion. Older age and better self‐rated health were associated with lower depersonalization. Finally, older age, having 4‐6 years of working experience in the current CCSC catchment area, and longer weekly working hours were linked to a lower score of reduced personal accomplishment.

**TABLE 3:** Contextual association among workplace social capital, community social capital, and burnout

	Emotional exhaustion				Depersonalization					Reduced personal accomplishment			
	Model 1		Model 2		Model 1			Model 2		Model 1		Model 2	
	*b* (SE)	*P*	*b* (SE)	*P*	*b* (SE)	*P*		*b* (SE)	*P*	*b* (SE)	*P*	*b* (SE)	*P*
Fixed effect													
Individual‐level													
Sex													
Men	−1.47 (0.33)	<.001	−1.35 (0.35)	<.001	0.37 (0.33)	.261		0.51 (0.36)	.159	−0.32 (0.34)	.347	0.02 (0.39)	.962
Age (every 10‐y increase)	−0.61 (0.14)	<.001	−0.64 (0.15)	<.001	−0.42 (0.14)	.002		−0.27 (0.15)	.071	−0.40 (0.14)	.003	−0.33 (0.15)	.029
Educational attainment													
Junior college/vocational school graduate or below	−0.31 (0.30)	.300	−0.31 (0.36)	.382	−0.14 (0.31)	.648		−0.21 (0.35)	.545	0.56 (0.31)	.074	0.50 (0.36)	.168
Self‐rated health													
Poor	2.90 (0.36)	<.001	2.69 (0.38)	<.001	1.50 (0.35)	<0.001		1.27 (0.37)	<.001	0.52 (0.37)	.159	0.28 (0.43)	.518
Sense of coherence	−0.23 (0.04)	<.001	−0.23 (0.05)	<.001	−0.15 (0.04)	<.001		−0.13 (0.05)	.011	−0.33 (0.04)	<.001	−0.34 (0.05)	<.001
Employment status													
Full‐time	−1.53 (0.48)	.001	−1.62 (0.56)	.004	−0.44 (0.39)	.255		−−0.67 (0.42)	.112	−0.19 (0.48)	.695	−0.50 (0.55)	.364
Managerial position													
Yes	0.60 (0.48)	.208	0.67 (0.49)	.177	0.73 (0.42)	.084		0.68 (0.42)	.107	0.18 (0.41)	.665	0.20 (0.43)	.641
Years of experience in the current catchment area of the CCSC (y)													
1‐3	−0.22 (0.36)	.536	−0.10 (0.40)	.796	−0.19 (0.32)	.550		0.09 (0.35)	.786	−0.40 (0.32)	.210	−0.39 (0.37)	.294
4‐6	−0.25 (0.39)	.510	−0.68 (0.41)	.099	0.28 (0.34)	.400		0.19 (0.35)	.594	−0.76 (0.37)	.038	−0.81 (0.40)	.046
Type of profession													
Certified social worker	0.34 (0.43)	.437	0.47 (0.53)	.368	0.16 (0.39)	.680		0.45 (0.45)	.317	0.46 (0.40)	.252	0.39 (0.46)	.396
Care manager/care worker	0.55 (0.39)	.160	0.51 (0.44)	.241	0.42 (0.32)	.189		0.21 (0.34)	.531	−0.09 (0.36)	.810	−0.14 (0.41)	.735
Average weekly working hours (every 10‐h increase)	0.36 (0.23)	.124	0.21 (0.27)	.436	0.23 (0.21)	.263		0.05 (0.22)	.807	−0.48 (0.20)	.017	−0.55 (0.23)	.016
Workplace social capital	−0.18 (0.03)	<.001	−0.18 (0.03)	<.001	−0.24 (0.03)	<.001		−0.26 (0.03)	<.001	−0.12 (0.02)	<.001	−0.12 (0.03)	<.001
Community social capital	−0.05 (0.04)	.211	−0.07 (0.04)	.123	−0.06 (0.03)	.101		−0.08 (0.04)	.049	−0.11 (0.03)	.002	−0.10 (0.04)	.015
Group‐level													
Workplace social capital			−0.18 (0.04)	<.001				−0.22 (0.03)	<.001			−0.07 (0.04)	.028
Community social capital			−0.04 (0.06)	.467				−0.11 (0.05)	.018			−0.17 (0.05)	.001
Proportion of people aged ≥ 65 y in the catchment area of the CCSC			−0.03 (0.05)	.634				0.02 (0.04)	.615			0.03 (0.04)	.506
Random effect													
Individual‐level variance (SE)	15.08 (0.14)		14.65 (0.13)		12.24 (0.12)			11.55 (0.12)		14.09 (0.13)		14.25 (0.13)	
Group‐level variance (SE)	2.32 (0.11)	<.001	2.04 (0.12)	<.001	1.56 (0.09)		<.001	0.84 (0.07)	<.001	0.61 (0.05)	.009	0.18 (0.03)	.131
Intra‐class correlation	13.3%		12.2%		11.3%			6.8%		4.1%		1.2%	

Note

Abbreviations: CCSC, Community Comprehensive Support Center; SD, standard deviation.

In Model 2, we additionally included the group‐level indicators to Model 1. Greater group‐level workplace social capital was significantly associated with lower scores of all three subscales (*b* = −0.18, −0.22, and −0.07 for emotional exhaustion, depersonalization, and reduced personal accomplishment, respectively), after adjusting for individual covariates, group‐level community social capital and aging rate of the community. Moreover, greater group‐level community social capital was significantly related to lower scores of the depersonalization and reduced personal accomplishment subscales (*b* = −0.11 and −0.17). Group‐level variance in Model 1 was explained: 11.9% for emotional exhaustion, 46.1% for depersonalization, and 7.0% for reduced personal accomplishment by adding the group‐level indicators in Model 2.

We additionally tested the interaction between the individual‐level covariates (ie individual characteristics and the CCSC‐related variables) and group‐level workplace/community social capital on the J‐MBI subscales in order to understand which factor could emphasize the effect of social capital on burnout. This analysis included a random slope in addition to a random intercept in the model. We found no significant interaction (data not shown in the table), which implied that the effects of the group‐level workplace and community social capital on burnout did not differ across the backgrounds of the participants.

## 4 DISCUSSION

The current study examined the relationship between group‐level workplace and community social capital and burnout among professionals working at the CCSCs in Japan, using multilevel analysis. There have been several previous works that tested the association between social capital, particularly workplace social capital, and burnout among various populations, including healthcare/welfare professionals.^24^, ^25^, ^26^, ^27^ However, these studies focused on individual‐level social capital. The analytic approach of this study, which regarded social capital as the group attribution, is distinct from that of previous studies. Therefore, it could provide novel insights into the contextual association of social capital on burnout.

Greater group‐level workplace social capital was associated with lower levels of burnout in all three dimensions. In addition to previous findings on the relationship between individual perception of workplace social capital (ie individual‐level workplace social capital) and burnout,^24^, ^25^, ^26^, ^27^ we confirmed that a socially cohesive workplace could have a beneficial influence on burnout condition of professionals working there. From the perspective of the widely accepted hypothesis that social capital affects health, people who belong to a highly cohesive workplace may find it easier to obtain social support from colleagues to cope with daily stress.^23^ This might decrease negative feelings, such as emotional exhaustion, toward their jobs. Moreover, this support can act as a source of self‐esteem and mutual respect within their workplace,^23^ which might, in turn, bring about higher personal accomplishment. Furthermore, some suggest that a community with high social capital has a function of informal social control, which is an ability to maintain social order or to intervene in deviant behaviors and attitudes.^35^ Thus, if one has a sign of depersonalization (eg cynical and unkind behaviors) then colleagues might be able to notice such features and intervene sooner.

We also found significant associations between higher group‐level community social capital and lower levels of depersonalization and reduced personal accomplishment. In communities showing high levels of social capital, it has been shown that information and knowledge can propagate more quickly (generally called social contagion),^36^ and people tend to access to healthcare services appropriately.^28^ In addition, social capital facilitates systematic and effective inclusions of community voices in the process of developing health and welfare policies/strategies,^37^ which can strengthen the efficacy of the CCSC activity. Therefore, professionals of CCSCs in such communities might be able to obtain more socioemotional rewards (eg personal validation and professional distinction) and feelings of creative achievement in their works, compared to those in communities with lower social capital, which might lead to lower depersonalization and higher personal accomplishment.

In addition to social capital, a higher SOC was associated with lower scores of all burnout subscales. This is consistent with other earlier studies.^38^, ^39^ SOC is a coping capability. People with a strong SOC tend to identify the nature of the particular stressor confronted and select the appropriate resources for a given situation.^40^ We confirmed that training to enhance a stress‐coping strategy of the workers could be effective in preventing their burnout.

The present study has some limitations. First, although the response rate was not low, there might be selection bias. For example, people with severe burnout would not participate in the survey, and people employed in a workplace with lower social capital were also unlikely to join the survey. This might have caused an underestimation of the association between social capital and burnout. Second, we created the group‐level social capital variables of both workplace and community by aggregating individual responses of the participants; however, multilateral assessment of social capital should be conducted to develop more genuine group‐level social capital indicators. In particular, the evaluation of community social capital by local residents would be useful in developing group‐level community social capital variables reflecting the reality of the community. Third, as this was a cross‐sectional study, we cannot discuss causality. Further investigation should be conducted to examine whether workplace and community social capital prevent deterioration in burnout longitudinally. Finally, the target community was limited to the central Tokyo area. Care should be taken when generalizing the findings.

## 5 CONCLUSION

This study revealed the contextual association of workplace and community social capital with burnout in professionals of health and welfare services for the seniors in Japan. We found that both group‐level workplace social capital and community social capital were associated with lower burnout. The current finding suggests that enhancing social capital of the workplace and community could be a possible strategy to prevent burnout among the health and welfare professionals.

## CONFLICT OF INTEREST

The authors declare no conflict of interest.

## AUTHOR CONTRIBUTIONS

HM, NK, MH, and YF conceived the ideas. HM, KN, and MH collected the data. HM analyzed the data and led the writing. NK, MH, and YF provided critical feedback. All authors approved the final manuscript.

## DISCLOSURE

*Approval of the research protocol:* The study protocol was reviewed and approved by the Ethical Committee of the Tokyo Metropolitan Institute of Gerontology. *Informed consent:* All participants were informed about the purpose of this study and that their participation was optional before inclusion in the study. This statement, a guarantee of anonymity, and other aspects of the cooperation requested were attached to the questionnaire. Return of the questionnaire was viewed as consent to participate in the survey. *Registry and the registration no. of the study/trial:* N/A. *Animal studies:* N/A.
